# Magnetite Nanoparticles and Essential Oils Systems for Advanced Antibacterial Therapies

**DOI:** 10.3390/ijms21197355

**Published:** 2020-10-05

**Authors:** Antonio David Mihai, Cristina Chircov, Alexandru Mihai Grumezescu, Alina Maria Holban

**Affiliations:** 1 Department of Science and Engineering of Oxide Materials and Nanomaterials, Faculty of Applied Chemistry and Materials Science, University Politehnica of Bucharest, 1–7 Gheorghe Polizu Street, 011061 Bucharest, Romania; antoniodavid.mihai@yahoo.com (A.D.M.); cristina.chircov@yahoo.com (C.C.); 2 Microbiology-Immunology Department, Faculty of Biology, University of Bucharest, 1–3 Portocalelor Lane, Sector 5, 77206 Bucharest, Romania; alina_m_h@yahoo.com or

**Keywords:** antibiotic-resistant bacteria, nanoscaled carriers, essential oils, magnetite nanoparticles, antibacterial nanotherapies

## Abstract

Essential oils (EOs) have attracted considerable interest in the past few years, with increasing evidence of their antibacterial, antiviral, antifungal, and insecticidal effects. However, as they are highly volatile, the administration of EOs to achieve the desired effects is challenging. Therefore, nanotechnology-based strategies for developing nanoscaled carriers for their efficient delivery might offer potential solutions. Owing to their biocompatibility, biodegradability, low toxicity, ability to target a tissue specifically, and primary structures that allow for the attachment of various therapeutics, magnetite nanoparticles (MNPs) are an example of such nanocarriers that could be used for the efficient delivery of EOs for antimicrobial therapies. The aim of this paper is to provide an overview of the use of EOs as antibacterial agents when coupled with magnetite nanoparticles (NPs), emphasizing the synthesis, properties and functionalization of such NPs to enhance their efficiency. In this manner, systems comprising EOs and MNPs could offer potential solutions that could overcome the challenges associated with biofilm formation on prosthetic devices and antibiotic-resistant bacteria by ensuring a controlled and sustained release of the antibacterial agents.

## 1. Introduction

Although most species of microorganisms are beneficial to nature and support life on Earth, a small group of infectious agents can cause severe diseases, sometimes leading to emerging outbreaks and difficult-to-handle pandemic situations. Pathogenic microorganisms hold the capacity to cause infections in the host by escaping the defense mechanisms through various strategies and virulence factors. According to The World Health Organization (WHO), bacteria, viruses, toxins, parasites, and harmful chemicals are listed as potential deadly biohazards [1,2,3]. Pathogenic bacteria may produce extracellular polymeric matrices, also known as glycocalyx, consisting of polysaccharides, lipids, and nucleic acids [1,4,5]. By these structures, bacteria adhere to biotic and abiotic substrates where they form biofilms, which are attached to the multicellular consortia of cells with particular architectures and modified phenotypes [1,5,6]. The formation of the biofilm is a primary protection strategy of microorganisms against environmental and antimicrobial agents, e.g., dehydration, starvation, antibiotics or biocides, but also host immune system [4]. Biofilm development is a fundamental factor in infection persistence or recurrence, with 65–80% associated being with chronic infections, according to The National Institutes of Health (NIH) [1,5,7].

The burden of biofilm development must be especially considered in the case of implantable medical devices, such as catheters, stents, prosthetic joints and implants, and internal and external fixation devices [8,9]. Considering the continuous increase in the use of such devices, with over tens of millions of patients worldwide and a market worth USD 74 billion in 2019, the associated infections are becoming a great concern as they can lead to significant morbidity, due to multiple surgeries, repeated antibiotic therapies and even amputation [8,9,10]. Currently, the main solution for preventing such complications is the prophylactic administration of systemic antibiotics and debridement [10,11]. However, this strategy is limited, as high amounts of antibiotics leads to organ toxicity and the development of antibiotic-resistant bacteria [8,10]. Additionally, the use of invasive devices can lead to the development of nosocomial infections, which are acquired between 48 h post-hospital admission and 3 days after discharge. According to WHO, at least 15% of the hospitalized patients are affected by these infections, of which the most common include urinary tract, surgical site, primary bloodstream, and wound infections, but also ventilator-associated pneumonia [12,13,14,15].

Ever since the discovery of penicillin in 1928 by Alexander Fleming and the consequent widespread and excessive use of antibiotics since the 1940s, associated antimicrobial resistance has arisen as a major concern, resulting in increased morbidity and mortality [16,17,18]. Therefore, antimicrobial-resistant bacteria, including *Pseudomonas aeruginosa*, *Staphylococcus aureus*, *Enterococcus faecalis*., *Klebsiella pneumoniae*, and *Enterobacter cloacae* as the major pathogens of concern, have become one of the 10 most critical public health problems, with 700,000 estimated deaths worldwide and 10 million deaths annually by 2050 [15,19,20,21]. In this manner, there is a fundamental need for developing novel technologies and global strategies to limit the spreading of infections and antimicrobial-resistant bacterial strains [22,23,24]. Recent studies focus on the discovery of non-antibiotic antibacterial agents that could attack multiple molecular targets, such as the binding and oxidizing of thiol groups within the chemical entities present onto the surface membrane of bacteria, blocking DNA replication, altering gene expression, denaturing enzymes, or inducing reactive oxygen species [18].

An increasing focus has been placed on essential oils (EOs), which are plant-derived secondary metabolites that play fundamental roles in defense mechanisms [25]. Since early times, EOs, a complex mixture of odoriferous and volatile organic compounds, have been used for a variety of medical purposes, including dermatology, cosmetics, and self-care natural medical products [26,27]. Additionally, there is increasing evidence of their general antimicrobial (i.e., antibacterial, antiviral, antifungal, and antiparasitic) effects, with many studies highlighting their potential for the treatment of resistant and difficult-to-treat infections [25,26,27]. However, because of their lipophilic and volatile nature, the administration of EOs in order to achieve the minimal inhibitory concentration against the bacterial pathogens is challenging. In this regard, microencapsulation and nanotechnology strategies could offer a potential solution [28]. Owing to their advanced stage of use and availability of commercial formulations, magnetic iron oxide nanoparticles are extensively studied for various applications, including drug delivery, magnetic resonance imaging, or theranostics [29,30]. Their applicability is related to the possibility of directing the nanoparticles at the desired site using magnetic fields, which will further induce heating and controlled release of the drugs. Furthermore, their magnetic properties have been proven to increase contrast and resolution in imaging techniques, thus improving diagnosis accuracy. Recent trends are focusing on merging the two directions of application, offering theranostic alternatives for both diagnosis and treatment. Iron oxide nanoparticles, especially magnetite, are highly advantageous due to their biocompatibility, biodegradability, non-toxicity, and ability to specifically target tissue and primary structures that allow for the attachment of various therapeutics [29,30,31,32,33,34]. Thus, magnetite nanoparticles (MNPs) and EOs systems could represent a novel and efficient direction in the management of infectious diseases.

In this regard, the aim of this paper is to provide an overview of the use of EOs as antibacterial agents, and the synthesis, properties, and functionalization of MNPs. Additionally, the most recent studies on MNPs and EOs systems for efficient antibacterial therapies will be highlighted.

## 2. Essential Oils as Antimicrobial Agents

As defined by the European Pharmacopeia 7th edition, EOs are “odorant products, with complex composition, obtained from raw plant extract, either extracted by a steam of water, dry distillation or a suitable mechanical method without heating” [35]. EOs comprise complex mixtures of more than 300 different volatile organic compounds, which are found in a partial vapor state at atmospheric pressure and room temperature [36]. Additionally, EOs are characterized by a lipophilic and hydrophobic nature and a density lower than that of water, which makes them highly soluble in organic solvents [37]. Among the large plethora of about 17,000 aromatic plant species, the most common EOs producing families are *Lamiaceae*, *Lauraceae*, *Poaceae*, *Asteraceae*, *Verbenaceae*, *Myrtaceae*, *Zingiberaceae*, *Rutaceae*, *Piperaceae*, *Cuppressaceae* and *Umbelliferae* [27,37].

Owing to their advantageous characteristics, EOs are receiving increasing interest in various fields, such as medicine, pharmaceutics, and cosmetics [27,37,38,39,40,41]. By 2022, it is expected that the EOs market will reach USD 11.67 billion, with Eco-SMART, the producer of various bioactive formulations, including Eugenol-Tween^®^ and Eugenol ethoxylate, ActiVin™, Pycnogenol^®^, and Herbalox^®^, as the world’s leading EO-based industry [42].

There are various extraction techniques for extracting EOs that use multiple plant segments, including seeds, buds, flowers, leaves, barks, and peels, with extraction yields depending on the species and segment type, solute and solvent properties, such as temperature, polarity, concentration, pH and acidity [37,43]. These techniques usually imply a physical method for avoiding any potentially significant change in the chemical composition of EOs [37]. Conventional EOs extraction methods include cold pressing, solvent extraction, enfleurage, and steam or water distillation. However, there are several disadvantages associated with these methods, such as high energy consumption and carbon dioxide emission [44]. In order to overcome these challenges and to enhance extraction yield and EOs quality, a novel promising “green” techniques that are non-thermal, cost-efficient, sustainable, and capable of producing EOs with similar or improved characteristics have been developed [42,43,44]. These techniques include supercritical fluid extraction, microwave-assisted extraction, ultrasound-assisted extraction, pressurized liquid extraction, accelerated solvent extraction, microwave steam distillation, and steam diffusion, pulse electric field extraction, enzyme-assisted extraction, molecular distillation, and instantaneous controlled pressure drop processes [42,43,45]. The advantages and disadvantages of the main extraction techniques are summarized in Table 1.

EOs are complex mixtures of volatile compounds, comprising two or three major components at relatively high concentrations, i.e., 20–70%, and 20–60 components (or up to 100) in trace amounts of different concentrations. Based on their hydrocarbon skeleton, the major components of EOs are classified into two structural families, namely terpenoids, such as carvacrol and thymol, and phenylpropanoids, such as cinnamaldehyde and eugenol. Based on the number of isoprene units, which are 5-carbon building blocks, terpenoids can be further sub-divided into monoterpenes, sesquiterpenes, and diterpenes, produced by the combination of two, three, or four units, respectively. Moreover, depending on the existence of ring structures, double bonds, oxygen addition, and stereochemistry, there are more than 1000 monoterpenes and 3000 sesquiterpenes. While both classes comprise phenolic compounds, their primary metabolic precursors and biosynthesis pathways are different. Specifically, terpenoids are synthesized via the mevalonate and mevalonate-independent pathways, while phenylpropanoids involve the shikimate pathways [28,38,46]. Furthermore, the volatile compounds found in EOs can be categorized based on their chemical classes, namely phenols, aldehydes, ketones, amines, amides, alcohols, and esters [25].

**Figure 1:**
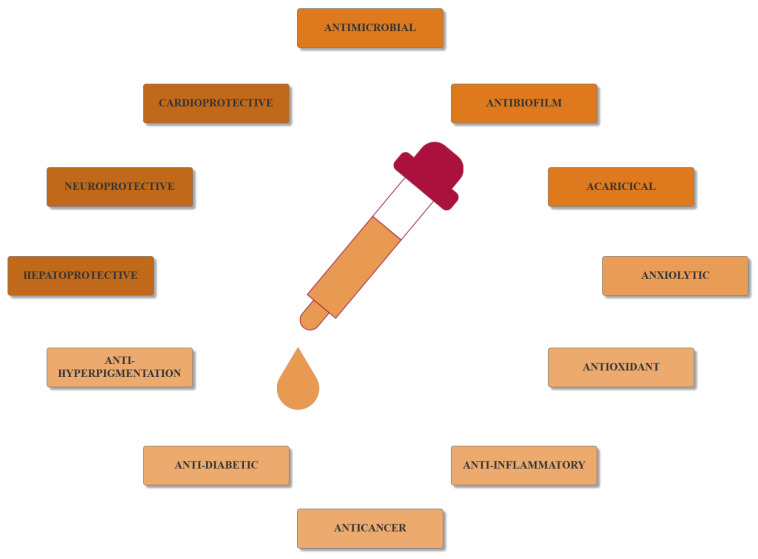
The main bioactive properties of EOs.

The therapeutic effects of EOs are manifested through the composition and concentration of these compounds. Therefore, they can exhibit a plethora of bioactive properties, including cardioprotective, hepatoprotective, neuroprotective, anxiolytic, antioxidant, anti-inflammatory, anticancer, anti-diabetic, anti-hyperpigmentation, antiviral, antifungal, antibacterial, antibiofilm, and acaricidal (Figure 1) [26,38,46,47].

Studies have shown the highest antibacterial activity for EOs containing carvacrol, eugenol, thymol, cinnamaldehyde, citral and linanaldehyde, and weaker activity for ketones or esters, such as β-myrcene, α-thujone or geranyl acetate. EOs containing terpene hydrocarbons are generally inactive [25,26]. The interactions between these compounds result in either enhanced or reduced antimicrobial efficiency, with four possible effect types, namely indifferent, additive, antagonistic, or synergistic. EOs’ antibacterial efficiency also depends on the bacteria type and their biochemical profile and ratio in crude [26,42].

**Figure 2:**
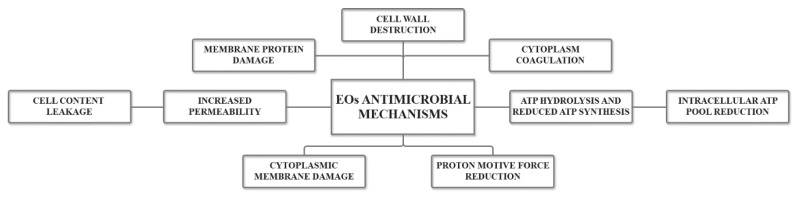
The main mechanisms involved in the antibacterial activities of EOs. Adapted from “Role of essential oils in food safety: antimicrobial and antioxidant applications” by Bhavaniramya et al., 2019 [25]. ATP = adenosine triphosphate.

Exhibiting both single and multiple target effects, EOs, owing to their hydrophobic nature, are capable of crossing the lipids constituting bacteria cell membranes and subsequently disrupting cell wall structures. In this manner, the increased membrane permeability causes electron flow, proton driving forces, active transport alterations, and cell content coagulation, and, consequently, ions and cellular materials leakage and cell death. The precise mechanisms involved in the antibacterial activity of EOs are illustrated in Figure 2 [25,42]. Additionally, EO antibacterial character is mostly provided by the presence of phenols, aldehydes, and alcohols within their composition [48].

Moreover, more than 60% of EO derivatives have exhibited varied antifungal effects, which could be of great importance in the food preservation industry [49]. The underlying mechanisms of EO antifungal activity are similar to the previously described antibacterial actions, namely membrane depolarization, proton pumps and ion channels disruptions, ATP pool depletion, and subsequent cell death by apoptotic and necrotic processes [50]. The antifungal bioactivity is generally given by the presence of phenolic compounds, including carvacrol, thymol, α-terpinyl acetate and cymene, terpenes, such as pinene and linalool, and the CHO group of unsaturated aldehydes, when conjugated with carbon in the form of C = C [49,50].

Generally, the antiviral activity of EOs is associated with virion envelopment interfering processes, which avert the entrance within the host cell. However, the underlying mechanisms are not completely understood, and further intensive research is required in the field [50].

Recent years have witnessed an incredible interest in the use of nanometric systems as carriers for EO delivery in antimicrobial applications [39,42]. In addition to offering protection against volatilization, nanomaterials have the advantage of increasing EO bioefficiency, as they are absorbed by cells and can penetrate membranes and biological barriers. However, as the synthesis of nanomaterials usually requires a heating or solvent evaporation step, the development of EOs and nanomaterials-based systems is still challenging [39]. MNPs have received considerable interest in the production of such systems, with many studies in recent years investigating the antimicrobial activity of MNPs and EOs systems.

## 3. Magnetite Nanoparticles—Synthesis, Properties, and Functionalization

Nanoparticles offer remarkable possibilities in terms of their electronic, optical, and magnetic properties, which have the potential to produce improved therapeutics and medical devices [51]. In this regard, inorganic nanoparticles have been widely considered as a platform for the development of tools for targeted drug delivery, clinical diagnostics, and medical imaging. The most common inorganic nanoparticles used for these purposes are iron oxide, silver, gold, zinc oxide, and titanium [52]. Among them, magnetic nanoparticles, the first generation of nanomaterials approved for clinical use, are particularly used owing to their superparamagnetic properties, which have increased the possibilities for developing novel and efficient biomedical applications [52,53,54], such as targeted drug and gene delivery, magnetic resonance imaging, biosensors, cancer detection and treatment, diagnosis and magnetic field-assisted radiotherapy, and tissue engineering [55]. The common types of iron oxide nanoparticles, which belong to the ferrimagnetic class of magnetic nanomaterials, are magnetite (Fe_3_O_4_), maghemite (γ-Fe_2_O_3_), hematite (α-Fe_2_O_3_), and mixed ferrites (MFe_2_O_4_, where M = Co, Mn, Ni or Zn) [52,53,56,57].

### 3.1. Synthesis Methods

**Figure 3:**
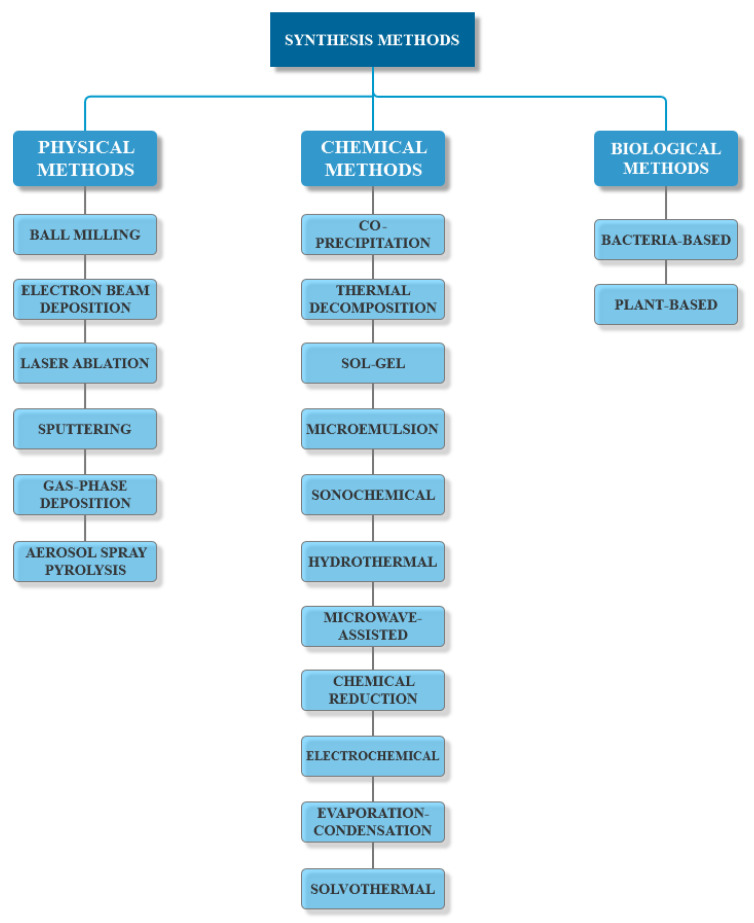
The main synthesis methods for the preparation of MNPs.

As their behavior strongly depends on their size, shape, and surface chemistry, MNP synthesis methods have been a hot research topic ever since their discovery. Moreover, any variation in the preparation process could subsequently lead to considerable changes in the final product. Therefore, considering the desired features and the field of application, there are three main routes for MNPs synthesis, namely physical, chemical, and biological methods (Figure 3) [55,58,59,60,61].

#### 3.1.1. Physical Methods

Physical methods for the preparation of MNPs follow a top-down approach in which bulk materials or large particles are broken into nanoscale particles. While such methods are preferred for the large-scale production of nanoparticles, there are major setbacks regarding uncontrolled particle size, non-uniform size distribution, and time-consuming and expensive technologies [31,62]. There is a variety of physical methods for synthesizing MNPs, including ball milling and electron beam lithography as the most commonly used, and others, such as laser ablation, sputtering, gas-phase deposition and aerosol spray pyrolysis [31,62,63]. On the one hand, the principle of ball milling, a solid-state synthesis method, is based on the generation of frictional force through the collision between the surfaces of the reactants, which causes an increase in temperature, pressure, and internal energy [64]. While this method is categorized into low energy and high energy milling, the latter is mostly preferred for the synthesis of nanoparticles [65]. On the other hand, electron beam lithography represents the conversion of high-purity bulk iron materials or films, which are deposited onto a substrate, to MNPs by emitting an electron beam across the material. Specifically, the electrons are focused and accelerated toward the materials, generating electron-electron collisions in the interaction volume of the incident electrons and modifying the chemical properties of the resistant layer. The initial iron precursors evaporate, and MNPs are subsequently formed [62,66].

#### 3.1.2. Chemical Methods

Chemical methods are the most commonly used, as they follow the bottom-up approaches, which are more efficient, easier to employ, and time and cost-efficient, and provide a narrower size particle distribution [59,67,68]. There are multiple chemical synthesis routes, and the most common are co-precipitation, thermal decomposition, sol-gel, and microemulsion [31,67,68].

The co-precipitation method is one of the most popular methods for MNPs synthesis, owing to its non-toxic nature, cost-efficiency, and mild reaction conditions [59,69]. It is based on the phenomenon through which a mixture of iron Fe^2+^ and Fe^3+^ salts in the form of nitrates, perchlorates, sulfates, and chlorides will precipitate at the addition of a base, such as ammonia, sodium, or potassium hydroxide, to form nanoparticles by nucleation and grain growth [59,68,70]. The use of polymers or surfactants is necessary to control particle size [70]. Furthermore, the size, shape, and magnetic properties of MNPs are influenced by the types and concentration of the salts, the pH and temperature of the medium, ionic strength, and mixing rate [59,70]. However, as MNPs obtained through co-precipitation have irregular shapes, wide size distribution, and low crystallinity, high-temperature synthesis methods are necessary to prepare high-quality MNPs [69,70].

In this manner, thermal decomposition is rapidly becoming one of the most common routes for the preparation of MNPs [69,71]. Through this method, MNPs are formed by the decomposition of iron precursors, such as acetylacetonates, acetates, or oleates, in high-boiling organic solvents containing surfactants and stabilizing agents, such as oleic acid and oleylamine. Moreover, the thermally unstable metal complexes are injected into the hot solution in order to generate a nucleation event followed by the controlled growth process. This allows for modifications of the synthesis parameters for tuning the size and shape of the nanoparticles [69,72].

The sol-gel method involves an initial solution of precursors, which are usually inorganic metal salts or metal-organic compounds in an adequate solvent. The addition of a surfactant will generate a polymerization reaction that will form the “sol”, which is a colloidal suspension of dispersed particles. Subsequently, a chemical treatment that will disable the surfactant will lead to the development of an extended network of linked particles throughout the solution, which will form the gel. The evaporation of the solvent will lead to the obtaining of MNPs [73]. While the sol-gel method is advantageous owing to its low cost, and the good homogeneity and high purity of the so-obtained nanoparticles, further heat treatments are required to reach a final crystalline state [69].

The microemulsion method implies the preparation of a microemulsion in an aqueous phase with ferric acid and/or ferrous ions, which will be precipitated through the injection of organic precipitating agents, such as cyclohexylamine or oleylamine [74]. While it offers the advantages of controllable size and shape, it also includes disadvantages related to aggregation and stability issues of the resulted MNPs [74,75].

Other techniques for the bottom-up synthesis of MNPs include sonochemical, hydrothermal, microwave-assisted, chemical reduction, electrochemical, evaporation-condensation, and solvothermal methods [31].

#### 3.1.3. Biological Methods

Biological methods involve green biosynthesis processes through bottom-up approaches. Specifically, metal precursors generate metal atoms that will assemble into nanoparticles. As the biological compounds present within the green substrates act as both reducing and capping agents, the final size, shape, and dispersibility can be controlled through a series of parameters, such as temperature, pressure, pH, and time of incubation [62]. The process involves the formation of small nucleation centers that grow through the sequestration of more metal ions around the nucleation site. Since they have high surface energies, the capping of the nanoparticles is necessary to prevent agglomeration and stabilize them [76]. These methods involve the use of bacteria, algae, fungi, and higher plants [31,62]. While biological methods are the most cost-effective and eco-friendly and generate the most biocompatible nanoparticles, publications within the field are still lacking [31].

Bacteria-based methods involve the use of magnetotactic and iron, reducing bacteria to produce MNPs intracellularly or extracellularly under anaerobic conditions. However, such methods usually necessitate further treatments, including calcination, sonication, and detergent usage, in order to eliminate the excess of capping biomolecules. By contrast, plant-based methods involve the use of various substrates, including callus, leaf, fruit, or seed extracts [62]. Plant-mediated MNPs synthesis represents the most efficient method for large-scale production in a short time. Additionally, waste products are easy to dispose of in the environment as they mostly comprise plant biomaterials [76].

### 3.2. Properties

Magnetite possesses unique physicochemical properties (Table 2), which further determine their interactions with biological tissues and, subsequently, their applicability in the biomedical field.

The size and shape of MNPs are the key characteristics that influence their pharmacokinetics, biodistribution, body clearance, and toxicity [78]. Based on their size, MNPs are classified into superparamagnetic iron oxide nanoparticles, ultrasmall superparamagnetic iron oxide nanoparticles, and a subset size of monocrystalline iron oxide nanoparticles [79,80]. Thus, size plays a fundamental role in the magnetic properties of MNPs and their subsequent response to magnetic fields. Specifically, as size decreases, the saturation magnetization decreases, and the nanoparticles exhibit supermagnetism [78]. Moreover, when applied in biomedicine, the size of MNPs should be <200 nm to avoid rapid spleen and liver filtration and to prolong blood circulation time, but >10 nm to avoid rapid kidney filtration [81]. Additionally, their size should be designed based on the type of biological entities they are intended for to promote cellular uptake, namely viruses (20–450 nm), proteins (5–50 nm), and cells (10–100 µm) [82]. MNPs have been synthesized in a variety of shapes, including nanospheres, nanocubes, nanowires, nanotubes, nanoplates, nanohexagons, nanooctahedrons, nanorods, nanorings, nanocapsules, and nanoflowers [81]. Their shape is influenced by the synthesis parameters, such as reactant concentration, reaction temperature, aging time, and interactions between the surface of the nanoparticles and the surfactants and/or capping agents [78].

Magnetite is a common iron oxide with a cubic inverse spinel structure and an Fd3m space group with a cell parameter of 8.394 Å. The 32 O^2−^ ions form ab fcc (face-centered cubic) closed packing structure, with Fe^2+^ ions disposed of in the octahedral sites, and Fe^3+^ ions disposed of both in the octahedral and tetrahedral sites [83,84,85]. The presence of the four unpaired electrons in the three-dimensional orbital of iron ions determines MNPs’ magnetic properties, which are measured through magnetization saturation, coercivity, and the magnetocrystalline anisotropy constant. Additionally, MNPs exhibit various individualities, such as superparamagnetism, saturation field, additional anisotropy contributions, and shifted loops subsequent to field cooling. Notably, superparamagnetism is the most important characteristic of MNPs, which is associated with particles of less than 20 nm in size. Such small and finite size, and surface effects, dominate the magnetic properties of MNPs, allowing for the free rotation of the particle in response to the magnetic field applied [70,86]. The interaction between MNPs and an external magnetic field will determine two processes, namely the orientation of the particle’s magnetic moment in a parallel direction to the magnetic field applied in order to minimize energy and bipolar interactions, and the transition of the particle in the gradient direction, as in magnetophoresis [77]. After removing the magnetic field, MNPs act with single-domain ferromagnetic properties and do not retain their magnetism, which consequently prevents physical aggregation. Size, shape, crystal lattice structure and size distribution directly influence the magnetic properties and behavior of MNPs [70].

When introduced into the organism, MNPs follow a series of steps, namely magnetic guidance to the target tissue or organ, immobilization and drug release through the external magnetic field, and standard clearance [78]. MNPs are a unique class of nanomaterials with well-proven superior biocompatibility in their native form that forms the basis for their widespread applicability in the biomedical field. By contrast to other biocompatible nanomaterials that are chemically and biologically inert, MNPs are catalytically active, exhibiting important physiological effects [87]. The cellular uptake of MNPs occurs through endocytosis, where they tend to accumulate in lysosomes and subsequently be degraded to iron ions. The toxicity of MNPs has been shown to be influenced by a series of factors, such as size, shape, configuration, and surface charge. Specifically, nanosized rod-shaped particles proved to be more toxic than microsized sphere-shaped particles. Furthermore, the toxicity of positively charged MNPs is increased as they undergo non-specific interactions and adsorptive endocytosis with negatively charged cell membranes [88]. An important aspect of the biological interactions of MNPs is microbial toxicity, which is due to a series of mechanisms, such as membrane depolarization and consequent cell integrity impairments, the release of iron ions that reach the nucleus and mitochondria and affect cellular homeostasis and protein coordination, the production of reactive oxygen species with lipid peroxidation, and DNA damage [88,89].

### 3.3. Functionalization

Simple MNPs are associated with issues related to their stability in colloidal solutions and biological fluids. First, as they are usually hydrophobic, positively charged, and have high surface energy, there is a high tendency to form large agglomerates through van der Waals and magnetic forces. Moreover, these agglomerates induce plasma protein adsorption, forming a protein corona on the surface that will favor rapid opsonization by the reticuloendothelial system or retention by the macrophages, and will reduce blood circulation half-life [70,77,78]. Second, their administration is associated with an increased risk of causing thrombosis and clots due to the occlusive effect of these agglomerates, which will hinder normal blood flow through the vessels [70].

In this regard, the surface modification of MNPs is a fundamental process for avoiding agglomeration and oxidation phenomena, such as Ostwald ripening, and improving stability and compatibility by providing interfacial electrostatic or steric repulsion forces between particles [77,78]. Additionally, surface functionalization enables the specific responses of nanoparticles to biological species and eliminates non-specific interactions with components within the system [90].

**Figure 4:**
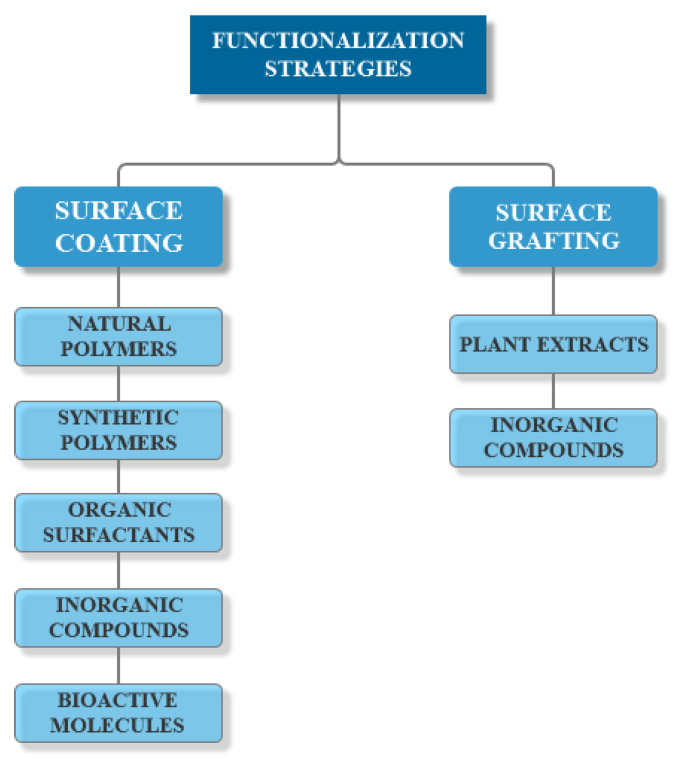
The main functionalization strategies for MNPs.

Advances in the area of materials science have allowed for facile surface tuning and functionalization strategies, which have increased the potential of MNPs in nanomedicine applications [91]. There are two main methods of functionalizing MNPs’ surfaces, either by surface coating or surface grafting (Figure 4) [31]. Surface coating is an effective way to prevent the dissolution of the nanoparticles, which further provides reactive functional groups for attaching various drugs, biomolecules, genes, or targeting ligands [78,90]. A variety of materials have been used for coating, including natural polymers (e.g., dextran, chitosan, pullulan, alginate, gelatin, agarose), synthetic polymers (e.g., polyethylene glycol, poly(ethylene-co-vinyl acetate), poly(vinylpyrrolidone), poly(lactic-co-glycolic acid), polyvinyl alcohol, polyacrylic acid, polyethyleneimine), organic surfactants, inorganic compounds, and bioactive molecules [70,77,78,88]. Surface grafting utilizes organic molecules, such as extracts and biomolecules extracted from plants, or inorganic compounds, such as silica, carbon, or metals (e.g., gold, cadmium, selenium, silver) [31,77]. Surface functionalization can be performed through two main techniques, namely during the synthesis process (in situ) by co-precipitation or microemulsion, or after finishing the synthesis process (ex-situ) through electrostatic attractions between the molecules and the surface of nanoparticles [31,70,77,90].

## 4. Antimicrobial Range of Essential Oils—Magnetite Nanosystems

Bacteria are widespread microorganisms generally grouped into genera and species depending on their morphological, biochemical, physiological, and genetic features. Specifically, they are categorized based on their shape, color, presence of flagella, growth ability onto certain media, and Gram staining [92]. Among them, Gram staining provides one of the most important classification systems, developed by Christian Gram in 1884. The approach relied on the cell envelope phenotypic characteristics, as some bacteria retain a crystal violet dye after ethanol discoloring, while others do not [93,94]. Thus, according to the Gram staining test, the bacteria which remain blue are called Gram-positive, while those that appear red are called Gram-negative. Such differences reflect variations in the cell wall structure [94]. With limited exceptions, Gram-positive bacteria possess a relatively simple cell wall of about 20–80 nm, comprising a single membrane surrounded by a thick layer of 30–200 molecules of peptidoglycans [93,94,95]. Additionally, the cell wall of Gram-positive bacteria contains water-soluble polymers of polyol phosphates, namely teichoic acids and lipoteichoic acids [94]. By contrast, Gram-negative bacteria have a more complex cell wall comprising a thin inner layer of less than 10 nm surrounded by a layer of one or two molecules of peptidoglycans, and an outer membrane containing the endotoxin lipopolysaccharide and a pore-containing structure [93,94,95]. Moreover, the space between the two membranes, known as the periplasm, contains a series of degradative enzymes and transport proteins [94]. The Gram staining classification is critically important as the characteristics of the cell envelope are associated with several cell properties, especially related to its response to outer stresses, such as ultraviolet radiation, heat or antibiotics [93].

Gram-positive bacteria are the most common causes of clinical infections, with approximately 60% arising from respiratory, intra-abdominal and urinary tract sources [96,97]. Including the *Staphylococcus*, *Streptococcus* and *Enterococcus* genera, Gram-positive bacteria are associated with diverse pathology spectra, varying from mild soft tissue infections to life-threatening systemic sepsis and meningitis [97,98].

*Staphylococcus aureus* is a Gram-positive bacterium that colonizes a considerable part of the human population, with 30% as a persistent carrier and 30% as an intermittent carrier, due to the abundant secreted and surface proteins that promote both colonization and immune response evasion. Specifically, tissue adhesion and host cell invasion occur through surface proteins, while immune response evasion and tissue damage are possible through the action of extracellular enzymes and zymogen activators. As most strains carry pathogenicity islands, prophages, transposons and insertion sequences, *Staphylococcus aureus* has the potential to cause various complications, from benign skin infections to osteomyelitis, endocarditis and septicemia [99,100]. Anghel et al. investigated the anti-adherence and antibiofilm properties of *Mentha piperita* EO using a 5 nm core–shell nanosystem based on MNPs. In this manner, the nanosystem was used for the surface modification of catheter devices. Due to the gradual release of the bioactive compounds within the EO, the antibiofilm properties of the surface coating remained, as the number of viable bacterial cells significantly decreased at 24, 48 and 72 h compared to the uncoated surface [101]. Rădulescu et al. developed a wound dressing biocompatible coating comprised of MNPs prepared through the co-precipitation of iron precursors in alkaline patchouli EO solutions. Results showed strong activities against *Staphylococcus aureus* biofilms maintained up to 72 h, with a decrease in viable biofilm-embedded cells of at least two-fold. Additionally, the systems exhibited low cytotoxicity on mammalian cells and good biodistribution after intraperitoneal injection in mice [102]. Moreover, Bilcu et al. performed a comparative study on the antimicrobial activity of MNPs functionalized with patchouli, vanilla, and ylang-ylang EOs as catheter coatings. Results showed the enhanced activity of the vanilla EO-based MNPs system, which inhibited both the initial *Staphylococcus aureus* cell adherence quantified at 24 h and the mature biofilm development quantified at 48 h [103]. Anghel et al. transferred 9.4 nm MNPs functionalized using *Cinnamomum verum* EO onto gastrostomy tubes through the matrix-assisted pulsed laser evaporation (MAPLE) technique. The uniform thin coating led to an increased surface biocompatibility, which allowed for a normal endothelial cell development up to 5 days and a significantly reduced *Staphylococcus aureus* colonization, with four-fold inhibition for incipient biofilms and three-fold for mature biofilms [104]. Furthermore, Negut et al. deposited *Nigella sativa* EO-functionalized MNPs and *Nigella sativa* EO-functionalized MNPs mixed with a polymeric matrix of poly(lactic-co-glycolic acid) on glass and silicone surfaces via the MAPLE technique. The results showed enhanced antimicrobial colonization and antibiofilm formation activities, and biocompatibility towards MG-63 cells, proving the potential use of such systems in the development of multifunctional antibiofilm medical surfaces [105]. Nutmeg EO was used by Mousavi et al. for improving MNPs′ saturation magnetization and antibacterial properties. Compared to the nutmeg ethanolic and methanolic essence, nutmeg EO-functionalized MNPs were shown to have superior antibacterial efficiency. Additionally, nutmeg EO-functionalized MNPs synthesized through a green method enhanced antimicrobial activity when compared to chemically synthesized MNPs [106]. Additionally, Grumezescu et al. developed eugenol-functionalized MNPs embedded into poly(3-hidroxybutyric acid-co-3-hidroxyvaleric acid)–polyvinyl alcohol microspheres through an oil-in-water emulsion technique. The microspheres were transferred through the MAPLE technique for obtaining thin antibacterial coatings. The microbiology tests demonstrated the inhibition of microbial adherence and biofilm development for *Staphylococcus aureus* at three time-points [107]. Similarly, Anghel et al. compared the microbicidal and anti-adherent properties of MNPs functionalized with eugenol and limonene. The results showed no significant differences when tested on *Staphylococcus aureus* strains, with the most important decrease in viable biofilm cells observed at 72 h, thus proving the ability of the nanosystem to combat the volatility of EOs when used for antimicrobial applications [108].

Enterococci are one of the main causes of nosocomial infections, which are responsible for both endemic and epidemic healthcare-associated infections [109,110]. *Enterococcus faecalis* is a Gram-positive coccus and facultative anaerobe, commonly found as a human intestinal commensal. Similar to other enterococci, *Enterococcus faecalis* is capable of producing oxygen-free radicals within the host, consequently causing chronic inflammation and colorectal cancer. Additionally, it has a high potential for spreading virulence and antibiotic-resistant genes through horizontal gene transfer [110,111]. The previously mentioned nutmeg EO-functionalized MNPs systems developed by Mousavi et al. exhibited a significant antibacterial character against *Enterococcus faecalis* strains [106].

Considering that Gram-negative bacteria exhibit increased levels of resistance towards most antibiotic classes, the infections caused by these microorganisms imply continuously increasing costs, morbidity, and mortality. Among them, *Escherichia coli*, *Pseudomonas aeruginosa*, *Acinetobacter baumannii*, and *Klebsiella pneumoniae* are the most common causes of health problems in hospitalized patients [112].

*Escherichia coli* is a remarkably diverse bacterial species, which is nearly ubiquitously found in the human gastrointestinal tract, despite accounting for a reduced portion of the gut microbiota. Although it does not generally compromise host health, virulence traits expressing strains can cause various diseases, such as urinary tract infections, through multiple mechanisms [113,114]. The thin coatings based on *Cinnamomum verum* EO-functionalized MNPs developed by Anghel et al. resulted in a slightly reduced *Escherichia coli* biofilm inhibition, ranging from 2.5-fold for initial biofilms and 2-fold for mature biofilms, which could demonstrate enhanced antimicrobial effects against Gram-positive bacteria [104]. Additionally, the *Nigella sativa* EO-functionalized MNPs and *Nigella sativa* EO-functionalized MNPs mixed with a polymeric matrix of poly(lactic-co-glycolic acid) exhibited similar results towards *Escherichia coli* biofilm, proving the potential of these systems for the prevention and/or treatment of Gram-negative bacteria infections [105]. The growth of *Escherichia coli* bacteria was also effectively inhibited in the presence of nutmeg EO-functionalized MNPs [106].

*Pseudomonas aeruginosa*, a Gram-negative bacillus, is an opportunistic pathogen ubiquitously found in nature, particularly in soil and water, characterized by a versatility that allows for its persistent survival under severe conditions [115,116]. While it does not usually cause diseases within healthy hosts, it is a major concern for patients with underlying immunocompromising conditions as it causes serious infections in the respiratory tract [115,117]. As its genome has provided knowledge of the mechanisms underlying the metabolism and pathology of this species, it has become the model for understanding microbial variations at the genome level and chronic disease evolution [115]. Tests on nutmeg EO-functionalized MNPs showed an efficiently inhibited growth of *Pseudomonas aeruginosa* bacterial strains [106]. The functionalization of MNPs with eugenol [107,108] and limonene [108] led to increased inhibiting effects on *Pseudomonas aeruginosa* biofilm formation and development. In the case of eugenol functionalization performed by Grumezescu et al., although the biofilm formation was significantly impaired in the first stages of development, the effect of the nanosystem decreased over time. This behavior could be associated with the greater antimicrobial effects against Gram-positive than Gram-negative strains of eugenol [107]. However, the results published by Anghel et al. stated no significant differences between Gram-positive and Gram-negative bacteria but demonstrated a higher antibiofilm inhibition when compared to limonene [108].

*Klebsiella pneumoniae*, an encapsulated Gram-negative bacterial strain, is widely found in the mouth, skin, and intestinal microbiota, but also in natural environments [118]. As it can cause both nosocomial and community-acquired pneumonia, *Klebsiella pneumoniae* is associated with high mortality rates in the bacteremia setting, mostly in the neonatal and infant populations [119]. The nanosystems developed by the authors of [103] proved no significant differences between patchouli, vanilla, and ylang-ylang EOs, exhibiting bioactivity mostly against *Klebsiella pneumoniae* adherence.

With approximately 90% of fungal infections, the five species most commonly associated with candidiasis are *Candida albicans* (65.3%), *Candida glabrata* (11.3%), *Candida tropicalis* (7.3%), *Candida parapsilosis* (6.0%), and *Candida krusei* (2.4%) [120]. *Candida albicans*, a human commensal and an opportunistic pathogen is the most commonly isolated species. While it is a normal part of mucosal microbiota found in the gastrointestinal, respiratory, and genitourinary tracts, *Candida albicans* is frequently responsible for nosocomial candidemia [120,121,122].

Saviuc et al. synthesized a core–shell system comprising MNPs and *Anethum graveolens* EOs. This method aids in the stabilization of EOs, further enhancing their antibiofilm activity against fungal adhesion on an inert substratum of the *Candida albicans*, *Candida tropicalis*, *Candida famata*, *Candida glabrata*, and *Candida krusei*. Results showed a decreased fungal adherence after 24 h of incubation, which increased after 48 h when compared to the untreated substratum [123]. Moreover, Grumezescu et al. used MNPs/oleic acid core–shell nanostructures for the stabilization of *Eugenia carryophyllata* EO on catheter surfaces. The use of the hybrid system based on eugenol and cariophylene-rich EOs significantly decreased biofilm development when compared to surfaces only treated with MNPs or EOs for *Candida albicans*, *Candida glabrata*, and *Candida krusei*. In some cases, biofilm development was totally inhibited after 24 h incubation [124]. Nanocoated wound dressings were obtained by Anghel et al. by submerging dressing pieces into nanofluids containing MNPs and *Satureja hortensis* EO. Scanning electron microscopy revealed the strong inhibition of *Candida albicans* adherence and consequent biofilm formation when compared to uncoated materials. Additionally, biofilm development was impaired both in early and mature phases, as quantified at 24, 48, and 72 h, which could be explained by the presence of phenolic compounds within the EO [125]. Similar results were also obtained in the case of wound dressings coated with MNPs functionalized with *Anethum graveolens* and *Salvia officinalis* EOs. Additionally, it was observed that the maximum intensity of inhibition was reached on the 48 h and 72 h biofilms, which could be explained by the different release rates [126]. Furthermore, Chifiriuc et al. investigated the effects of *Rosmarinus officinalis* EO-functionalized MNPs against the *Candida albicans* and *Candida tropicalis* clinical strains. The results showed considerably higher inhibitory effects for nanosystem-treated catheters, with yeast cells practically absent at 48 and 72 h for both strains [127]. Antifungal adherence and antibiofilm properties were also observed when using the *Cinnamomum verum* EO-functionalized MNPs developed by Anghel et al. [104], and the *Nigella sativa* EO-functionalized MNPs and *Nigella sativa* EO-functionalized MNPs mixed with a polymeric matrix of poly(lactic-co-glycolic acid) systems developed by Negut et al. [105].

Table 3 summarizes all the nanosystems based on MNPs and EOs that have been described above for antimicrobial therapies.

## 5. Conclusions and Future Perspectives

Recent studies have focused on the discovery of non-antibiotic antimicrobial agents that could attack multiple molecular targets, such as by blocking DNA replication, altering gene expression, denaturing enzymes, inducing reactive oxygen species, or damaging microbial membranes. Owing to the increasing evidence of their antibacterial, antiviral, antifungal, and insecticidal effects, EOs have attracted tremendous interest in recent years for a variety of applications in the fields of medicine, pharmaceutics, and cosmetics. Additionally, the hydrophobic nature of EOs allows for crossing the lipids constituting bacteria cell membranes and subsequently disrupting cell wall structures, thus inducing promising antibacterial effects. However, as environmental factors might cause EO degradation and/or loss of bioactivity due to volatilization, there has been increasing interest in the use of nanometric systems as carriers for EOs delivery. As they are highly advantageous, owing to their biocompatibility, biodegradability, non-toxicity, ability to specifically target tissue, and primary structures that allow for the attachment of various therapeutics, MNPs represent a potential candidate for the development of EO-based nanosystems for antibacterial therapies. In this manner, promising solutions that could overcome the challenges associated with biofilm formation on prosthetic devices and antibiotic-resistant bacteria could be developed. While the current state of research focuses mainly on the antibacterial and antifungal purposes of such nanosystems, future works should also focus more on developing antiviral and antiparasitical applications, as each individual component has proven to be efficient.

## Figures and Tables

**Table 1:** The advantages and disadvantages of the main EOs extraction techniques [44,45].

Extraction Method	Advantages	Disadvantages
Cold Pressing	Lower Temperatures Used During the Process	Low Yield;
Solvent Extraction	Lower Temperatures Used During the Process; Inexpensive; Fast	Trace Amounts Of Solvent; Low Yield; Loss of Volatile Compounds; Long Extraction Times
Enfleurage	Lower Temperatures Used During the Process	Time-Consuming; Labor-Intensive; Expensive;
Steam or Water Distillation	Straightforward Process	High Temperatures Which Can Cause Chemical Changes in the Compounds; Loss ff Volatile Compounds; Low Yield; Might Require Oil Rectification (Redistillation)
Supercritical Fluid Extraction	Low Temperatures Used During The Process; High Selectivity; Environmentally Friendly; Analytically and Industrially Scalable; Fast; Low Energy Costs	Lower EOs Quantities
Microwave-Assisted Extraction	Reasonable Costs; Good Performance under Atmospheric Conditions; Higher Extraction Yields; Shorter Extraction Times	Requires Higher Quantities of Organic Solvent; Less Environmentally Friendly
Ultrasound-Assisted Extraction	Minimum Effects on Extractable Compounds; Reduction/Avoidance of Organic Solvents; Shorter Extraction Times; High Yields	Expensive
Instantaneous Controlled Pressure Drop Process	Enhanced Global Diffusivity; Does Not Require the Use of Solvents; Higher Extraction Yields; Lower Energy Required; No Chemical Changes in the Compounds	-

**Table 2:** A summary of the main physicochemical properties of magnetite [77].

Molecular Formula	Color	Density	Melting Temperature	Hardness	Type of Magnetism
Fe_3_O_4_	black	5.18 g/cm^3^	1583−1597 °C	5.5	Ferrimagnetic
Curie Temperature	Saturation Magnetization at 300 K	Standard Gibbs Free Energy of Formation	Crystallographic System	Structure Type	Lattice Parameter
858 K	92–100 A∙m^2^/kg	−1012.6 kJ/mol	cubic	inverse spinel	Å = 0.8396 nm

Where, Å—Lattice constant.

**Table 3:** A summary of the MNPs and EOs nanosystems for antimicrobial therapies.

Microbial Species	Type of Nanosystem	EO Main Constituents	Nanosystem Dimension	MIC Value	Desired Application	Reference
*Staphylococcus aureus*	Core–Shell MNPs Functionalized with *Mentha piperita* EO	n.r.	5 nm	n.r.	Catheter Coating	[101]
	MNPs Functionalized with Patchouli EO	n.r.	7.5 nm	n.r.	Wound Dressing Coating	[102]
	MNPs Functionalized with Patchouli, Vanilla, and Ylang Ylang EOs	n.r.	>20 nm	n.r.	Catheter Coating	[103]
	MNPs Functionalized with *Cinnamomum verum* EO	n.r.	9–10 nm	n.r.	Gastrostomy Tube Coating	[104]
	MNPs Functionalized with *Nigella sativa* EO and MNPs Functionalized with Nigella Sativa EO Embedded into a Polymeric Matrix of Poly(lactic-co-glycolic acid)	n.r.	14–21 nm	0.62 mg/mL	Antimicrobial Coatings	[105]
	MNPs Functionalized with Nutmeg EO	Limonene, Terpineol, Carvacrol, and Thymol	10–15 nm	250 μg/mL and 125 μg/mL	Antimicrobial Coatings	[106]
	MNPs Functionalized with Eugenol	-	>10 nm	n.r.	Antimicrobial Coatings	[107]
	MNPs Functionalized with Eugenol And Limonene	-	10 nm	n.r.	Wound Dressing Coating	[108]
*Enterococcus faecalis*	MNPs Functionalized with Nutmeg EO	Limonene, Terpineol, Carvacrol, And Thymol	10–15 nm	250 μg/mL and 125 μg/mL	Antimicrobial Coatings	[106]
*Escherichia coli*	MNPs Functionalized with *Cinnamomum Verum* EO	n.r.	9–10 nm	n.r.	Gastrostomy Tube Coating	[104]
	MNPs Functionalized With *Nigella Sativa* EO And MNPs Functionalized With *Nigella Sativa* EO Mixed With A Polymeric Matrix Of Poly(Lactic-Co-Glycolic Acid)	n.r.	14–21 nm	1.25 mg/mL	Antimicrobial Coatings	[105]
	MNPs Functionalized with Nutmeg EO	Limonene, Terpineol, Carvacrol, and Thymol	10–15 nm	62.5 μg/mL and 31.25 μg/mL	Antimicrobial Coatings	[106]
*Pseudomonas aeruginosa*	MNPs Functionalized with Nutmeg EO	Limonene, Terpineol, Carvacrol, and Thymol	10–15 nm	125 μg/mL and 62.5 μg/mL	Antimicrobial Coatings	[106]
	MNPs Functionalized with Eugenol	-	>10 nm	n.r.	Antimicrobial Coatings	[107]
	MNPs Functionalized with Eugenol And Limonene	-	10 nm	n.r.	Wound Dressing Coating	[108]
*Klebsiella pneumoniae*	MNPs Functionalized with Patchouli, Vanilla, And Ylang-Ylang Eos	n.r.	>20 nm	n.r.	Catheter Coating	[103]
*Candida albicans*	Core-Shell MNPs Functionalized with *Anethum Graveolens* EO	Limonene, Carvone, and A-Phellandrene	n.r.	n.r.	Antimicrobial Coatings	[123]
	Core-Shell MNPs Functionalized with *Eugenia Carryophyllata* EO	n.r.	>20 nm	n.r.	Catheter Coating	[124]
	MNPs Functionalized with *Satureja Hortensis* EO	Carvacrol, γ-Terpinene, p-Cymene, α-Terpinene, and Myrcene	10 nm	n.r.	Wound Dressing Coating	[125]
	MNPs Functionalized with *Anethum Graveolens* And *Salvia Officinalis* Eos	cis-thujone, eucalyptol, α,β-pinene, and camphene	13–17 nm	n.r.	Wound Dressing Coating	[126]
	MNPs Functionalized with *Rosmarinus Officinalis* EO	Eucalyptol, Camphor, Caryophyllene, and A-Pinene	>20 nm	n.r.	Catheter Coating	[127]
	MNPs Functionalized with *Cinnamomum Verum* EO	n.r.	9–10 nm	n.r.	Gastrostomy Tube Coating	[104]
	MNPs Functionalized With *Nigella Sativa* EO And MNPs Functionalized With *Nigella Sativa* EO Mixed With A Polymeric Matrix Of Poly(Lactic-Co-Glycolic Acid)	n.r.	14–21 nm	0.62 mg/mL	Antimicrobial Coatings	[105]
*Candida tropicalis*	Core-Shell MNPs Functionalized with *Anethum Graveolens* EO	limonene, carvone, and α-phellandrene	n.r.	n.r.	Antimicrobial Coatings	[123]
	MNPs Functionalized with *Rosmarinus Officinalis* EO	eucalyptol, camphor, caryophyllene, and α-pinene	>20 nm	n.r.	Catheter Coating	[127]
*Candida famata*	Core-Shell MNPs Functionalized with *Anethum Graveolens* EO	limonene, carvone, and α-phellandrene	n.r.	n.r.	Antimicrobial Coatings	[123]
*Candida glabrata*	Core-Shell MNPs Functionalized with *Anethum Graveolens* EO	limonene, carvone, and α-phellandrene	n.r.	n.r.	Antimicrobial Coatings	[123]
	Core-Shell MNPs Functionalized with *Eugenia Carryophyllata* EO	n.r.	>20 nm	n.r.	Catheter Coating	[124]
*Candida krusei*	Core-Shell MNPs Functionalized with *Anethum Graveolens* EO	limonene, carvone, and α-phellandrene	n.r.	n.r.	Antimicrobial Coatings	[123]
	Core-Shell MNPs Functionalized with *Eugenia Carryophyllata* EO	n.r.	>20 nm	n.r.	Catheter Coating	[124]
*Candida parapsilosis*	MNPs Functionalized with *Cinnamomum Verum* EO	n.r.	9–10 nm	n.r.	Gastrostomy Tube Coating	[104]

Where, n.r.—not related; “-” not applicable.
